# Validation of days alive and out of hospital as a new patient-centered outcome to quantify life impact after heart transplantation

**DOI:** 10.1038/s41598-022-21936-4

**Published:** 2022-11-01

**Authors:** René M’Pembele, Sebastian Roth, Alexandra Stroda, Tilman Reier, Giovanna Lurati Buse, Stephan U. Sixt, Ralf Westenfeld, Philipp Rellecke, Igor Tudorache, Markus W. Hollmann, Hug Aubin, Payam Akhyari, Artur Lichtenberg, Ragnar Huhn, Udo Boeken

**Affiliations:** 1 Department of Anesthesiology, Medical Faculty and University Hospital Duesseldorf, Heinrich-Heine-University Duesseldorf, Düsseldorf, Germany; 2 Department of Cardiology, Pulmonology and Vascular Medicine, Medical Faculty and University Hospital Duesseldorf, Heinrich-Heine-University Duesseldorf, Düsseldorf, Germany; 3 Department of Cardiac Surgery, Medical Faculty and University Hospital Duesseldorf, Heinrich-Heine-University Duesseldorf, Moorenstr. 5, 40225 Düsseldorf, Germany; 4 Department of Anesthesiology, Amsterdam University Medical Center (AUMC), Location AMC, Amsterdam, The Netherlands; 5 Department of Anesthesiology, Kerckhoff Heart and Lung Center, Bad Nauheim, Germany

**Keywords:** Risk factors, Quality of life, Outcomes research, Heart failure

## Abstract

The number of patients waiting for heart transplantation (HTX) is increasing. Thus, identification of outcome-relevant factors is crucial. This study aimed to identify perioperative factors associated with days alive and out of hospital (DAOH)—a patient-centered outcome to quantify life impact—after HTX. This retrospective cohort study screened 187 patients who underwent HTX at university hospital Duesseldorf, Germany from September 2010 to December 2020. The primary endpoint was DAOH at 1 year. Risk factors for mortality after HTX were assessed in univariate analysis. Variables with significant association were entered into multivariable quantile regression. In total, 175 patients were included into analysis. Median DAOH at 1 year was 295 (223–322) days. In univariate analysis the following variables were associated with reduced DAOH: recipient or donor diabetes pre-HTX, renal replacement therapy (RRT), VA-ECMO therapy, recipient body mass index, recipient estimated glomerular filtration rate (eGFR) and postoperative duration of mechanical ventilation. After adjustment, mechanical ventilation, RRT, eGFR and recipient diabetes showed significant independent association with DAOH. This study identified risk factors associated with reduced DAOH at 1-year after HTX. These findings might complement existing data for outcome of patients undergoing HTX.

## Introduction

Orthotopic heart transplantation (HTX) is a complex procedure which is carried out at specialized centers^1^. As the number of patients waiting for HTX is constantly increasing, efficient perioperative resource management and a careful selection of donors and suitable patients for HTX are highly relevant^2^. To optimize perioperative resource management, the identification of outcome-relevant perioperative factors in HTX patients is crucial. Previous studies tried to identify factors associated with poor outcome and mostly focused on hard endpoints such as mortality^3,4^. A high-quality meta-analysis by Foroutan et al. investigated influence of different recipient-, donor- and transplant-associated variables on 1-year mortality after HTX^5^. Donor and recipient age, creatinine concentration, mechanical ventilation, recipient diabetes and mechanical circulatory support were identified to be significantly associated with 1-year mortality in HTX patients^5^.

Recently, more patient centered outcomes have been investigated in HTX patients as traditional mortality analysis might be insufficient to measure life impact. Days Alive and Out of hospital (DAOH) is a statistically efficient patient-centered outcome to measure life impact of a procedure^6,7^. Further advantages of DAOH are that it is easy to measure, readily available and it can be regarded as a composite of multiple clinically relevant outcomes including mortality, length and number of (re-)hospitalization and—indirectly—health care costs due to hospitalizations. A previous study showed that veno-arterial extracorporeal membrane oxygenation (VA-ECMO) therapy due to primary graft dysfunction has critical life impact at 1-year after HTX measured by DAOH^8^. Interestingly, patients who could be successfully weaned from VA-ECMO showed lower DAOH as compared to patients without primary graft dysfunction whilst 1-year mortality did not differ between groups in this study. This finding illustrates utility of DAOH and emphasizes clinical importance of this outcome beyond traditional endpoints.

Beside VA-ECMO therapy further important factors might be associated with reduced DAOH in HTX patients. However, evidence on prognostic factors for DAOH after HTX is very limited. Identification of those risk factors is crucial to complement survival data. Therefore, the present study aimed to identify donor-, recipient- and procedure-related prognostic variables for DAOH at 1-year after HTX.

## Patients and methods

### Study design and ethical statement

This retrospective single-center cohort study was conducted in compliance with the Declaration of Helsinki, guidelines for good clinical practice (GCP) and the International society for Heart and Lung Transplantation (ISHLT) ethics statement. Ethical approval for this retrospective study was obtained on 25th of January 2021 from the University of Duesseldorf’s ethics committee (reference number: 4567). All patients gave written informed consent to be registered in a local prospective HTX database in the past so that the ethics committee waived the need for additional written informed consent for this retrospective analysis. The “Strengthening the Reporting of Observational Studies in Epidemiology” (STROBE) guidelines were used for standardized reporting of the study results^9^.

### Participants

All consecutive patients aged ≥ 18 years who underwent HTX at the University Hospital Duesseldorf, Germany from September 2010 to December 2020 were included. Patients with missing data and incomplete medical records regarding the primary endpoint were excluded.

### Outcome assessment

DAOH at 1 year after HTX was the primary endpoint of this study. Calculation of DAOH was performed as previously described^8,10^. In brief, all days of hospitalization in the first year after HTX were summed up and subtracted from 365 days. Outpatient visits and emergency department visits not exceeding 24 h were excluded from DAOH analysis. In case of mortality within the first year after HTX, days the patient did not survive were added to days of hospitalization before subtracting them from 365 days. Notably, DAOH does include the time spent in cardiac rehabilitation centers or similar institutions patients were transferred to after hospital discharge. All HTX patients are closely connected to our center so that external hospitalizations without our knowledge are very unlikely.

### Data collection

Data of patients were derived from the continuously updated local prospective HTX database and the patient’s electronic medical records. These data consisted of patient characteristics, comorbidities, information on treatment and complications during hospital stay, as well as date of mortality and days of hospital stay during the first year after HTX.

### Identification of included variables

We primarily based the choice of variables on a meta-analysis by Foroutan et al. which summarized risk factors for 1-year mortality after HTX^5^. We considered all variables which were included into the final forrest-plot, regardless of significant association with 1-year mortality. Selected variables which were available in our prospective HTX database were included into analysis. Accordingly, the following 19 predefined recipient-, donor- and transplant-related variables were included: (1) preoperative variables: recipient age, recipient sex, underlying disease, recipient diabetes, recipient estimated glomerular filtration rate (eGFR), recipient arterial hypertension, recipient body mass index (BMI), recipient pulmonary hypertension, recipient cytomegalovirus (CMV) status, previous cardiothoracic surgery, left ventricular assist device before HTX, donor age, donor sex, donor diabetes, sex mismatch between donor and recipient, total ischemic time; (2) postoperative: recipient renal replacement therapy (RRT), duration of mechanical ventilation, use of veno-arterial extracorporeal membrane oxygenation (VA-ECMO). Data on influence of VA-ECMO therapy on DAOH has been published previously by our group^8^. The present study complements these data, as sample size was smaller in the previously published report.

### Statistical analysis

Statistical analysis was performed in GraphPad Prism© version 8.02 (La Jolla, California, USA) and IBM SPSS© software version 25.0 (Armonk, NY, USA). Patient characteristics were presented as mean ± standard deviation (SD) or as median and interquartile ranges (IQR, 25–75%), as appropriate, for continuous variables and numbers (n) with corresponding percentages (%) in brackets for categorical variables. Boxplots were created for categorical variables to visualize DAOH and Mann–Whitney-U-test was used to compare DAOH between groups. Continuous variables were categorized into quartiles or according to international classifications if feasible and presented as boxplots. Association between continuous variables and DAOH was analyzed using Kruskal–Wallis test. Variables with significant association with DAOH in univariate analysis were included in a multivariable model. For multivariate analysis, we chose a quantile regression model which accounts for non-linear associations between independent variables and DAOH as dependent variable. In this model, all percentiles of DAOH were investigated. At each level the associations of independent variables with DAOH were investigated in a multivariable model. We predefined that factors affecting DAOH in 10th and 20th DAOH percentile as relevant based on the current literature^7^. These quantiles represent patients with the lowest DAOH from the total patient cohort. According to our statistical protocol, we entered following variables into our multivariable quantile regression model: Recipient diabetes, RRT, ECMO, Donor DM, recipient BMI, Recipient eGFR and duration of mechanical ventilation. Sensitivity analysis was performed for univariate analysis by excluding all patients who died during the first year after HTX. For all results of statistical analysis, a *p*-value < 0.05 was considered as significant.

## Results

In the time period from September 2010 to December 2020, 187 patients underwent HTX at the University hospital Duesseldorf, Germany. According to the inclusion and exclusion criteria, 12 patients had to be excluded as DAOH could not be computed. Therefore, 175 HTX patients were included into our analysis. Mean age was 54 ± 11 years and 134 patients (76.6%) were male. Detailed patient characteristics are presented in Table 1. Median DAOH at 1 year was 295 days (IQR 223–322). Reasons for rehospitalization are presented in Table 2. Overall, 32 patients (18.3%) died during the study period. Eleven patients died out of hospital from unknown causes. In-hospital causes of death were: sepsis (8 patients), intracranial hemorrhage (3 patients), mesenteric ischemia (3 patients), graft failure (2 patients), cerebral hypoxia (3 patients), bleeding (1 patient) and multiple organ failure (1 patient).

**Table 1:** Characteristics of HTX recipients and donors.

	HTX patients (N = 175)
**Baseline characteristics recipients mean ± S.D. or No. (%)**	
Age (years)	54 ± 11
BMI (kg/m^2^)	25.6 ± 4.6
male	134 (76.6)
Creatinine (mg/dl)	1.4 ± 1.0
**Underlying disease**	
ICM	69 (39.4)
DCM	93 (53.1)
ARVC	6 (3.4)
HCM	3 (1.7)
others	4 (2.3)
**Preoperative conditions**	
Arterial hypertension	105 (60.0)
Pulmonary hypertension	18 (10.3)
Diabetes mellitus	37 (21.1)
Cytomegalovirus IgG status	101 (57.7)
LVAD	92 (52.6)
Previous cardiothoracic surgeries	114 (65.1)
**Postoperative conditions**	
Mechanical ventilation (h)	151 ± 13
RRT	102 (58.3)
VA-ECMO	52 (29.7)
Length of hospital stay (d)	46 ± 35
Length of ICU stay (d)	25 ± 28
**Donor characteristics**	
Age (years)	43 ± 13
BMI (kg/m^2^)	25.9 ± 3.9
male	101 (57.7)
Sex mismatch	51 (29.1)
diabetes	12 (6.9)
LVEF (%)	59 ± 12
History of CPR	42 (24.6%)
Length of CPR (min)	15 (9–21)
**Intraoperative conditions**	
Total ischemic time (min)	218 ± 51
Duration of surgery (min)	445 ± 116
**Outcome**	
DAOH	295 (223–322)
1-year mortality	32 (18.3)
Mean survival (d)	313 ± 116
Numbers of hospital readmissions	3 (1–4)
Time from HTX to death (d)	36 (17–115)

*BMI* Body mass index, *ICM* ischemic cardiomyopathy, *DCM* dilated cardiomyopathy, *ARVC* arrhythmogenic right ventricular cardiomyopathy, *HCM* hypertrophic cardiomyopathy, *LVAD* left ventricular assist device, *RRT* renal replacement therapy, *VA-ECMO* veno-arterial extracorporeal membrane oxygenation, *DAOH* days alive and out of hospital.

**Table 2:** Reasons for hospital admission and corresponding mean days of hospital stay.

Reason for hospital admission	No. of patients	Days of hospital stay (mean ± S.D.)
HTX	175	48 ± 39
Endomyocardial biopsy	126	7 ± 4
Gastrointestinal disorders	15	14 ± 12
Respiratory infections	18	20 ± 14
Wound infection/impaired wound healing	14	25 ± 30
Urinary tract infections	5	16 ± 18
Other infections	22	23 ± 20
Acute kidney injury	13	10 ± 5
Graft rejection reaction	24	16 ± 18
Bleeding complications	6	21 ± 12
Hematological disorders	3	16 ± 13
Epileptic seizure	3	14 ± 10
Non-cardiac surgery	14	13 ± 12
Other reasons	10	25 ± 34

*HTX* Heart transplantation.

### Univariate association of categorical variables with DAOH

**Figure 1:**
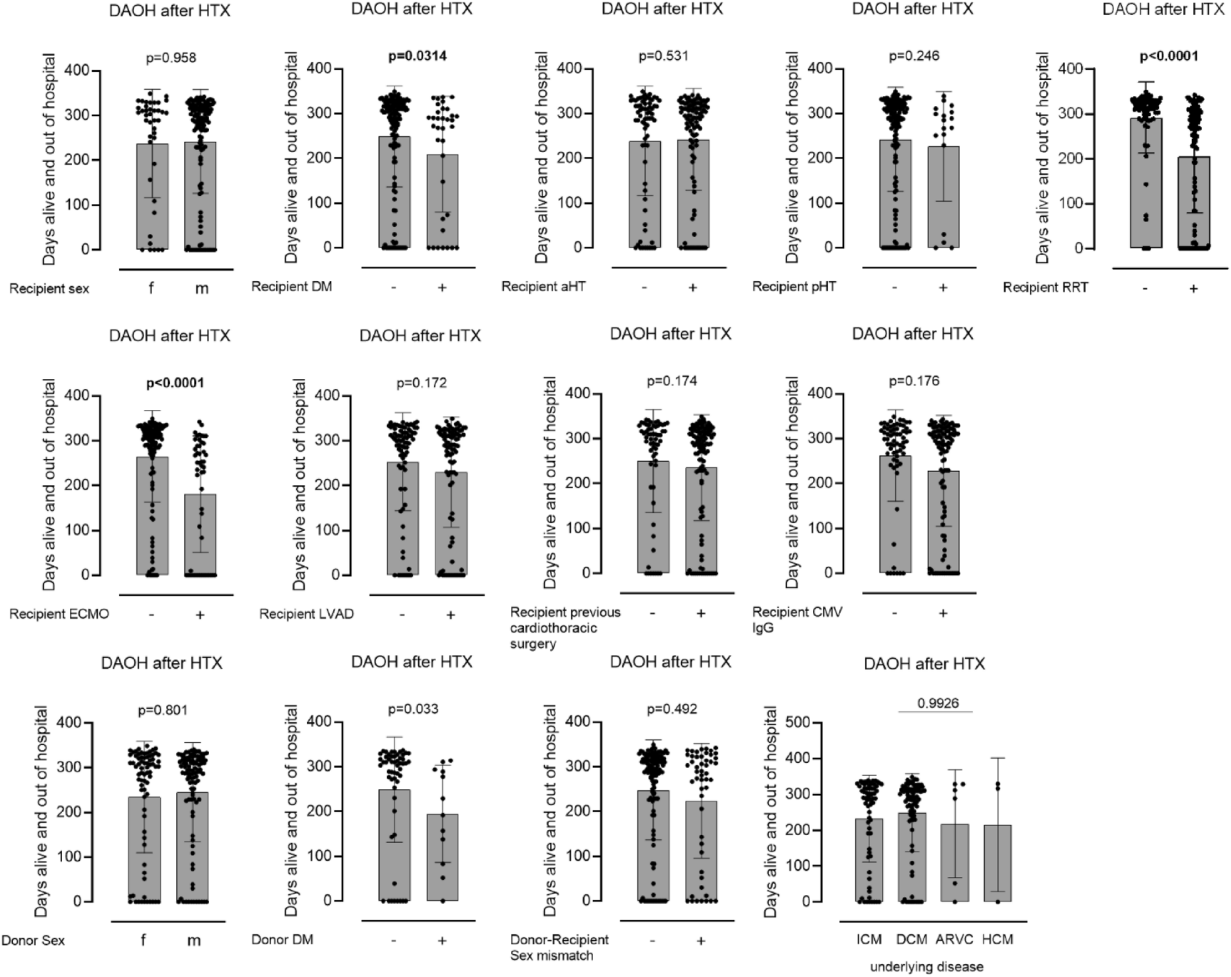
Influence of categorical variables on DAOH. Univariate analysis for the association of 13 categorical variables with days alive and out of hospital (DAOH) at 1 year after heart transplantation (HTX).

After univariate analysis of the 13 categorical variables, four variables were significantly associated with DAOH. As preoperative factors we identified recipient and donor diabetes to be associated with lower DAOH [recipient diabetes: 303 (247–323) days vs. 272 (97–293) days *p* = 0.0314; donor diabetes: 308 (229–323) days vs. 211 (65–303) days *p* = 0.0329]. As postoperative variables, renal replacement therapy (RRT) and VA-ECMO therapy were identified [RRT: 316 (295–329) days vs. 267 (75–305) days *p* =  < 0.0001; VA-ECMO: 309 (273–327) days vs. 243 (0–290) days *p* =  < 0.0001] (Fig. 1, Table 3).

**Table 3:** Variables investigated for association with days alive and out of hospital in univariate analysis.

Categorical variables	Total No. of patients	No. of patients per subgroup	Univariate association with DAOH
Recipient sex	175	Male: 134	No
		Female: 41	
Recipient diabetes	175	Yes: 35	Yes
		No: 140	
Recipient arterial hypertension	175	Yes: 105	No
		No: 70	
Recipient pulmonary hypertension	175	Yes: 18	No
		No: 157	
Recipient RRT	175	Yes: 102	Yes
		No: 73	
Recipient ECMO	175	Yes: 50	Yes
		No: 125	
Recipient LVAD	175	Yes: 92	No
		No: 83	
Recipient previous cardiothoracic surgery	175	Yes: 114	No
		No: 61	
Recipient CMV status	175	Positive: 110	No
		Negative: 65	
Donor sex	175	Male: 101	No
		Female: 74	
Donor diabetes	66*	Yes: 12	Yes
		No: 54	
Donor-recipient sex mismatch	175	Yes: 51	No
		No: 124	
Underlying disease	171**	ICM: 69	No
		DCM: 93	
		ARVC: 6	
		HCM: 3	
Continuous variables Recipient age	175	01:44	No
		02:43	
		03:51	
		04:37	
Recipient BMI	175	01:08	Yes
		0.1382	
		0.1681	
		04:26	
Recipient eGFR	175	01:40	Yes
		02:53	
		03:40	
		04:42	
Duration of mechanical ventilation	175	01:41	Yes
		02:43	
		03:43	
		04:48	
Total ischemic time	175	01:43	No
		02:44	
		03:45	
		04:43	
Donor age	175	01:43	No
		02:39	
		03:49	
		04:44	

*BMI* Body mass index (kg/m^2^), *CMV* cytomegalovirus, *ECMO* extracorporeal membrane oxygenation, *eGFR* estimated glomerular filtration rate, *LVAD* left ventricular assist device, *RRT* Renal replacement therapy.

*Missing data from donors’ hospital.

**4 Missing patients with other underlying diseases.

### Univariate association of continuous variables with DAOH

**Figure 2:**
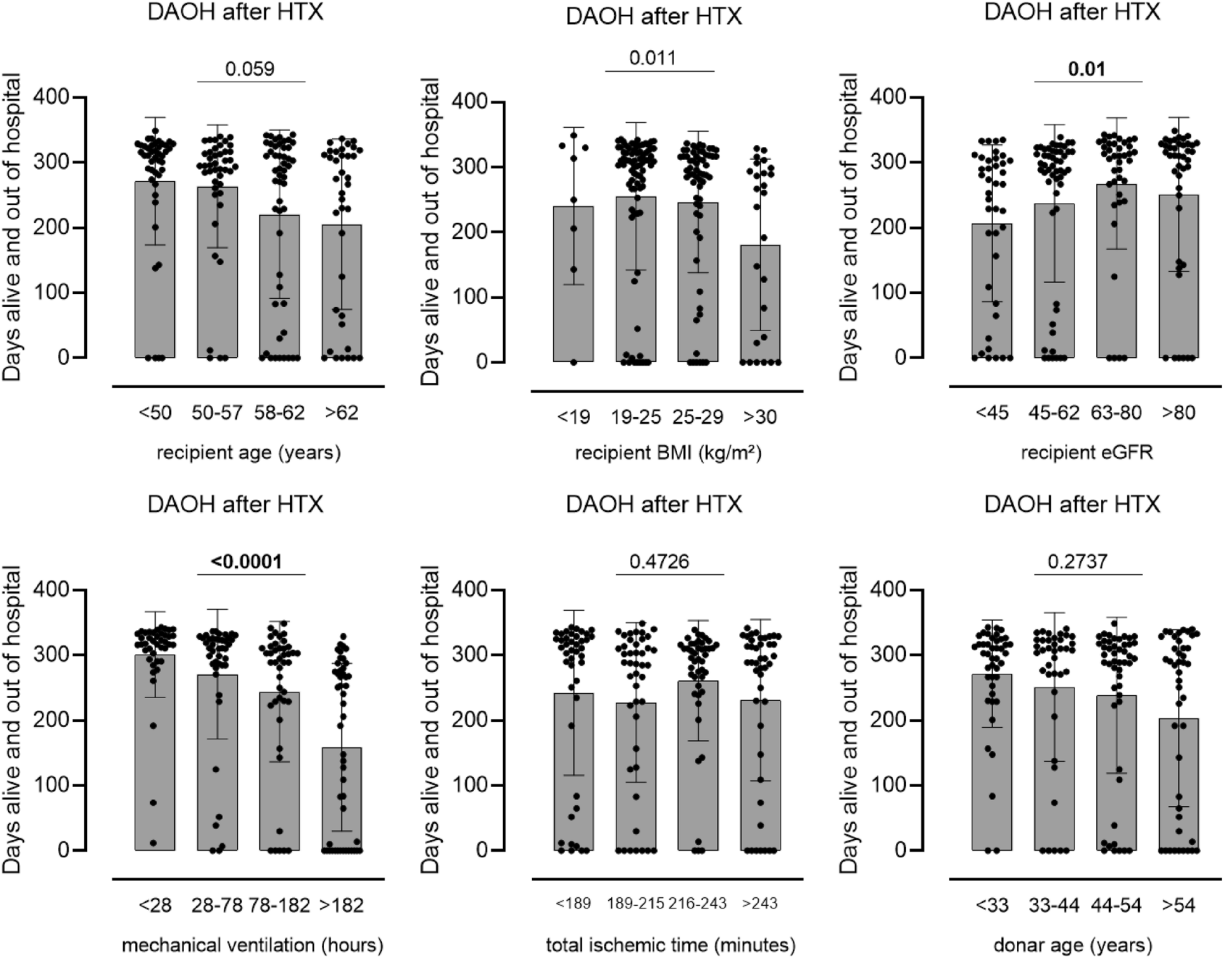
Influence of continuous variables on DAOH. Univariate analysis for the association of 6 continuous variables with days alive and out of hospital (DAOH) at 1 year after heart transplantation (HTX). To visualize distribution of DAOH, all continuous variables were stratified by quartiles.

Association of 6 prespecified continuous variables with DAOH was investigated by using non-parametric Kruskal–Wallis test for column comparison, after variables were stratified by median and IQR or international classification. Out of these variables, recipient eGFR, recipient BMI and postoperative duration of mechanical ventilation were significantly associated with lower DAOH [recipient eGFR: < 45 ml/min = 260 (90–303) days vs. 45-62 ml/min = 289 (226–317) days vs. 63-80 ml/min = 310 (255–329) days vs. > 80 ml/min = 311 (210–329) days; *p* = 0.01; recipient BMI: < 19 kg/m^2^ = 282 (159–332) days vs. 19–25 kg/m^2^ = 308 (253–327) days vs. 25–29 kg/m^2^ = 290 (228–317) days vs. > 30 kg/m^2^ = 250 (23–295) days; *p* = 0.011 mechanical ventilation: < 28 h = 318 (299–334) days vs. 28–78 h = 311 (285–326) days vs. 78–182 h = 289 (229–311) days vs. > 182 h = 199 (0–277) days; *p* =  < 0.0001]. Kruskal–Wallis test for column comparison did not show significant difference between columns for donor and recipient age regarding DAOH, despite clear visual trend (Fig. 2, Table 3).

### Independent association of variables with DAOH in multivariable analysis

**Figure 3:**
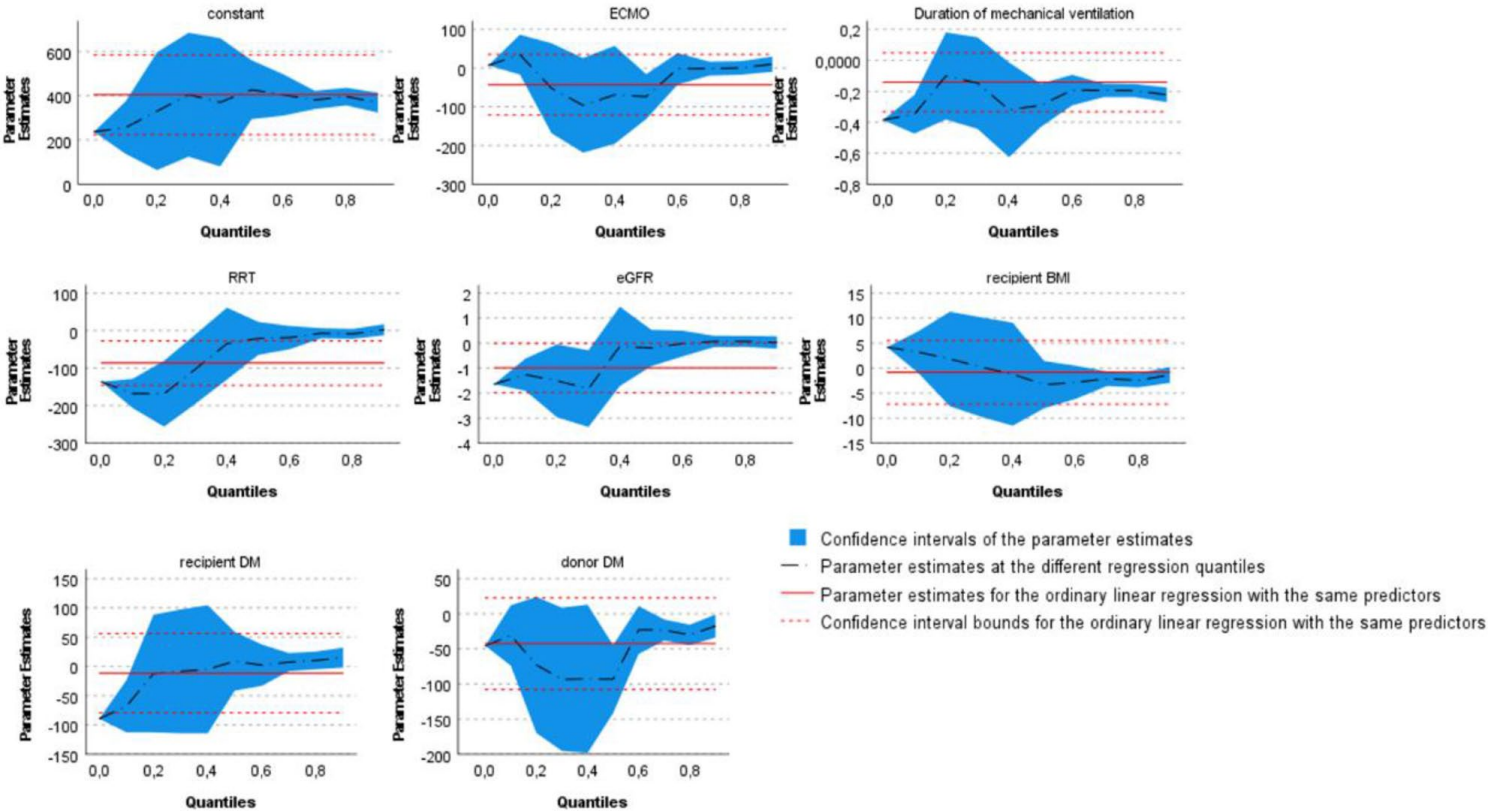
Association of variables with DAOH in multivariable quantile regression model. The figure shows the influence of selected variables on DAOH in a multivariable quantile regression model. Y-axis shows DAOH estimates while X-axis shows different quantiles. The black line represents parameter estimates at different regression quantiles. The Confidence intervals of quantile regression are presented in blue. Red lines represent parameter estimates and confidence interval of an ordinary linear regression with same variables. Duration of mechanical ventilation, renal replacement therapy (RRT), estimated glomerular filtration rate (eGFR), recipient diabetes mellitus (DM) were significantly associated with lower DAOH in the 10th and 20th percentile in this quantile regression model.

All variables which were significantly associated with DAOH in univariate analysis were included into a multivariable quantile regression model and the 10th and 20th percentile of this model were investigated for association of variables with DAOH. The pseudo-R^2^ of the final model reached from 0.39 to 0.41 for the selected quantiles, indicating a moderate goodness of fit. In this model recipient diabetes, recipient eGFR, duration of mechanical ventilation and postoperative RRT had independent impact on DAOH in patients with low DAOH (Fig. 3).

### Sensitivity analysis without 1-year mortality

To ensure that our findings in univariate analysis of risk factors and DAOH were not mainly influenced by 1-year mortality we performed a sensitivity analysis. In this analysis we excluded all patients who died during the first year after HTX and reanalyzed univariate association of risk factors and days out of hospital. Most of the significant associations remained after analysis except from donor diabetes, recipient age and donor age. For detailed results please see supplementary figures (Figs. S1 and S2).

### Independent association of preoperative variables with DAOH

We additionally performed the quantile regression model including only preoperative parameters (recipient BMI, recipient eGFR, donor diabetes and recipient diabetes). In this model, only donor and recipient diabetes showed influence on DAOH in the 10th and 20th percentile of the model*.* However, model performance was poor. Detailed results are presented in the supplements (Fig. S3).

## Discussion

This study aimed to identify recipient-, donor- and procedure-related risk factors which might have an influence on postoperative outcome at 1-year after HTX as measured by DAOH. We could identify that recipient diabetes, donor diabetes, RRT, VA-ECMO therapy, recipient BMI, recipient eGFR and postoperative duration of mechanical ventilation were associated with lower DAOH according to univariate analysis. Only recipient diabetes, recipient eGFR, mechanical ventilation and postoperative RRT remained independently associated with reduced DAOH after multivariate analysis.

### Variables associated with mortality within the first year after HTX

A previous meta-analysis of Foroutan et al. investigated variables associated with 1-year mortality in patients after HTX, as mortality is highest in the first year after surgery in these patients. This meta-analysis included results of 62 studies including 282.367 HTX patients. Recipient age, congenital etiology of heart failure, recipient diabetes, kidney function, dialysis, mechanical ventilation, mechanical circulatory support, donor age, and sex mismatch (especially transplantation from female donor to male recipient) were identified to be associated with 1-year mortality in this study^5^. This meta-analysis served as a basis for the present analysis.

### Variables associated with reduced DAOH within the first year after HTX

In the present study we could confirm that recipient diabetes, RRT and mechanical ventilation have a significant and independent life impact as measured by DAOH. In our study cohort we did not show significant influence of other variables, e.g. congenital heart failure. This is likely due to the fact that we did only include 6 patients with arrhythmogenic right ventricular cardiomyopathy (ARVC), 3 patients with hypertrophic cardiomyopathy (HCM) and no patients with other congenital heart failure as underlying diseases into our analysis. Therefore, sample size is very limited to show any effect on DAOH, in line with the findings for 1-year mortality, there was no difference in DAOH between patients representing with ischemic and dilated cardiomyopathy^5,11–13^. We could also not show an association for sex mismatch which might again be related to our limited sample size as a visual trend can be seen within boxplots. Interestingly, we showed in our sensitivity analysis of the endpoint that at least some risk factors (eGFR, VA-ECMO, RRT, mechanical ventilation, donor diabetes) showed significant impact on DAOH independent from 1-year mortality, while impact of other risk factors was mainly driven by 1-year mortality. This underlines the strength of the endpoint as it combines both, days out of hospital and 1-year mortality and therefore might be more sensitive to measure life impact as sole mortality analysis as described previously^7,14^.

### Risk factors which showed association with DAOH but not with mortality

Notably, we identified a univariate association between donor diabetes and reduced DAOH in our study. This is interesting as findings of two previous studies show contradicting results concerning influence of donor diabetes on 1-year mortality^15,16^. However, within the meta-analysis no influence could be detected^5^. As those results are based on only two studies and data is very limited, quality of this result is indicated as moderate. In this case DAOH might be a more sensitive parameter to measure life impact. A previous study in HTX patients could show that mortality analysis and DAOH analysis can differ significantly^8^. In cases of donor diabetes this might reflect that patients had longer hospital stay without increased mortality. Additionally, we could identify in our study that patients with BMI ≥ 30 kg/m^2^ had lower DAOH as compared to patients with BMI < 30 kg/m^2^ according to univariate analysis. In a previous study BMI was identified to be associated with mortality in female recipients but not in male recipients^17^. Another data registry study including 38.498 patients showed that underweight and obese patients had significantly higher risk for mortality^18^. In this context another recent registry study confirmed the results for obese patients^19^. However, previous evidence on influence of BMI on 1-year mortality is contradicting as other studies showed similar 1-year survival of obese patients compared to normal BMI patients^13,20,21^.

Regarding the results of our analysis, we can see that there was a visually clear trend for some variables without showing statistical significance. E.g. the DAOH in recipients receiving donor hearts < 33 years appear relevantly lower than the DAOH in recipients receiving donort hearts > 54 years. From a clinical/patient point of view, this difference might be of importance as recipients receiving older donor hearts may have similar (or even lower) DAOH in comparison with LVAD patients. First data suggest that older patients initially might benefit more from LVAD implantation, than from older donor hearts which consequently raises further interesting and relevant questions^22^. In this context, it is important to mention that the sample size in this study was rather small so that several results might become statistically significant when increasing the sample size. Therefore, our data can only serve as a first basis for further investigations. In the future, DAOH as a new patient-centered measure after HTX may be implemented in existing registries so that more data will be available to complement and validate the findings of this study.

### Strengths and limitations of this study

This was a retrospective single-center cohort study with a limited sample size, limiting the external applicability of the results. However, most patient characteristics and outcomes of our cohort correspond to the current literature and thus may be regarded as representative. As mentioned above, this study included only a selected number of predefined variables. The choice was based on a meta-analysis to ensure adequate choice of variables. Hence, it is possible that not all risk factors with influence on DAOH were included into this study. To cover as many relevant variables as possible, we performed analyses for further variables not included in the meta-analysis. The results can be found in the supplements (Fig. S4). A strength of this study is the endpoint DAOH which might be more suitable to measure life impact of recipient, donor and transplant associated risk factors as compared to mortality. We also showed in our sensitivity analysis that most variables not only have impact on 1-year mortality but also on hospital stay and hospital readmissions. We performed a 365-day follow-up in this study. Due to the retrospective nature of the study, we cannot guarantee that every hospitalization was reported within this year. However, HTX patients are closely connected to our center. Therefore, it is very unlikely that these patients were hospitalized at another hospital without our knowledge within the first year after HTX. Finally, it is important to mention that the results of this analysis regarding postoperative VA-ECMO support differ from our previously published work^8^. This discrepancy can be explained by the fact that the sample size as well as the choice of covariables were different between both works.

## Conclusions

This study identified recipient- and donor-associated risk factors with influence on DAOH—a more patient centered outcome to quantify life impact after HTX. Our findings support previous evidence for mortality analysis and complement the existing data. Our results may help to improve perioperative resource management and to optimize careful donor and recipient selection of patients undergoing HTX. Further studies with a prospective design are needed to validate our results in a variety of larger cohorts.

## Supplementary Information

Supplementary Information.

## Data Availability

All relevant data are included in the present manuscript or in the supplements. Raw data are available upon reasonable request by the first author R.M.

## Abbreviations

ARVC: Arrhythmogenic right ventricular cardiomyopathy
BMI: Body mass index
CMV: Cytomegalovirus
DAOH: Days alive and out of hospital
eGFR: Estimated glomerular filtration rate
GCP: Good clinical practice
HCM: Hypertrophic cardiomyopathy
HTX: Heart transplantation
IQR: Interquartile range
RRT: Renal replacement therapy
SD: Standard deviation
VA-ECMO: Veno-arterial extracorporeal membrane oxygenation
