# Supplementary material for: Validation of days alive and out of hospital as a new patient-centered outcome to quantify life impact after heart transplantation
Source: Sci Rep. 2022 Nov 1;12:18352. doi: 10.1038/s41598-022-21936-4 (PMC9626454; doi:10.1038/s41598-022-21936-4)
Supplement: Supplementary file 1 — Supplementary Information. [file 41598_2022_21936_MOESM1_ESM.docx]

**Supplementary material**

**Figure S1: Influence of categorical variables on days out of hospital**


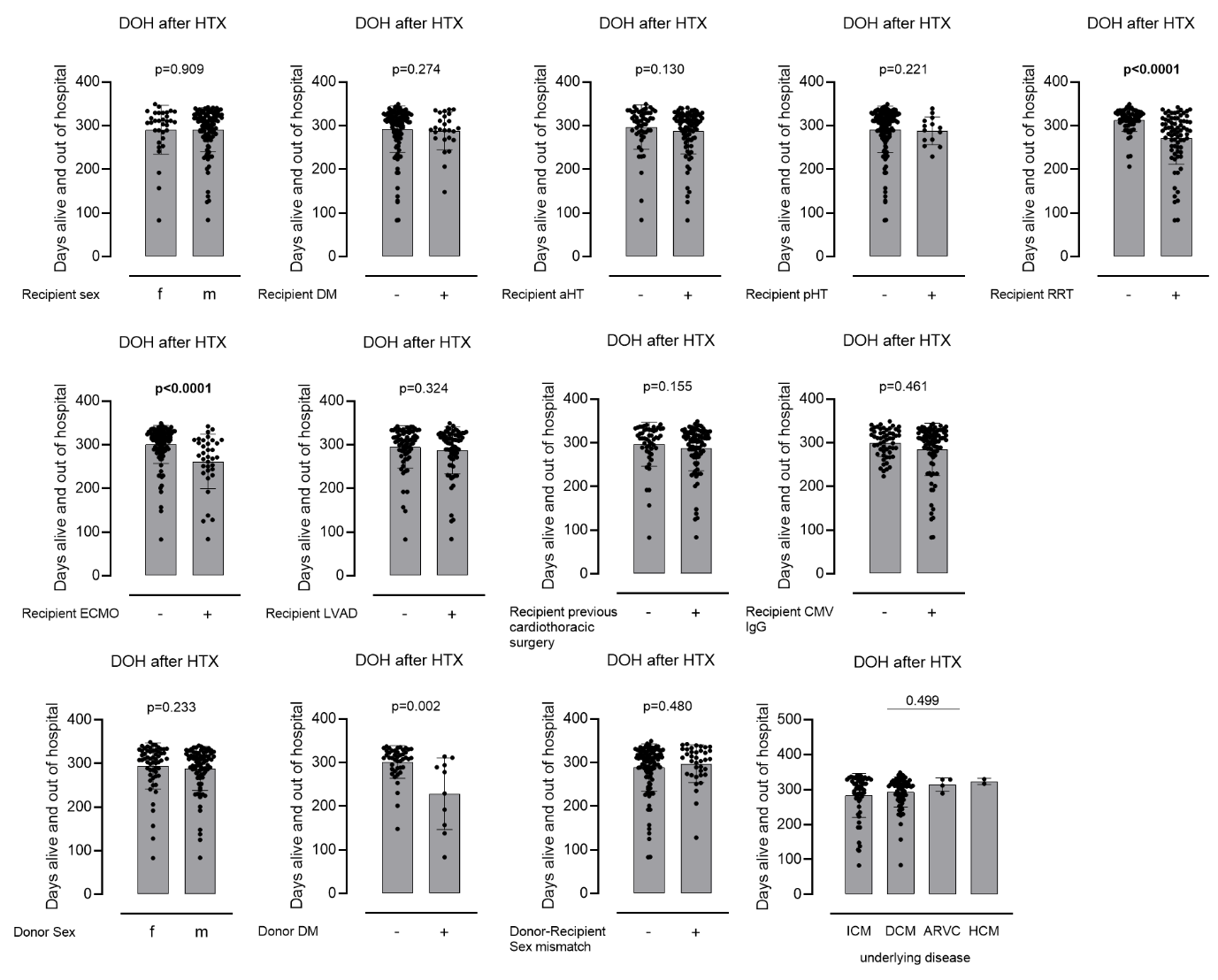


**Legend:** Univariate analysis for the association of 13 categorical variables with days out of hospital (DOH) at 1 year after heart transplantation (HTX) after excluding patients who died.

**Figure S2: Influence of continuous variables on days out of hospital**


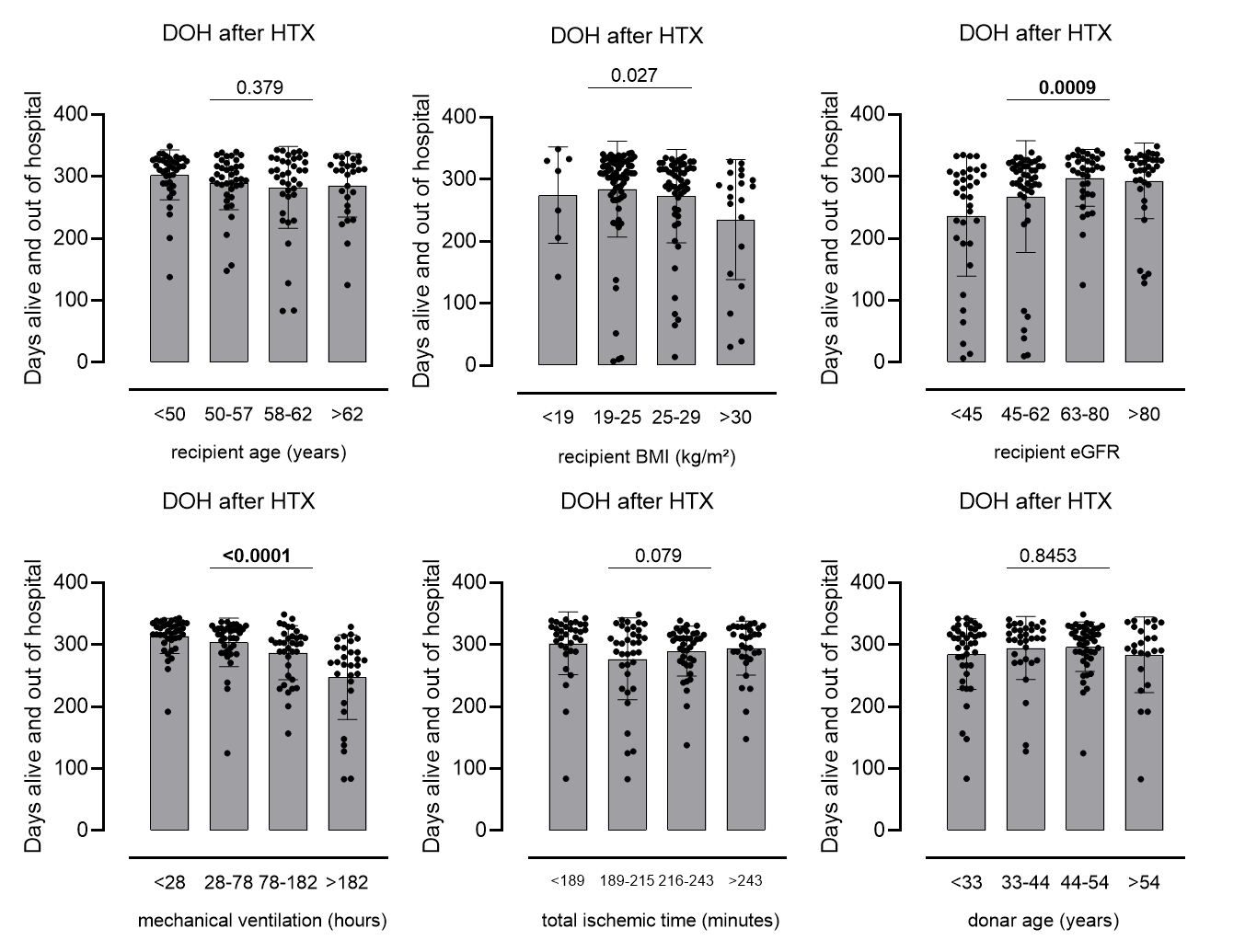


**Legend:** Univariate analysis for the association of 6 continuous variables with days out of hospital (DOH) at 1 year after heart transplantation (HTX) after excluding patients who died.

**Figure S3: Association of preoperative variables with DAOH in multivariable quantile regression model.**


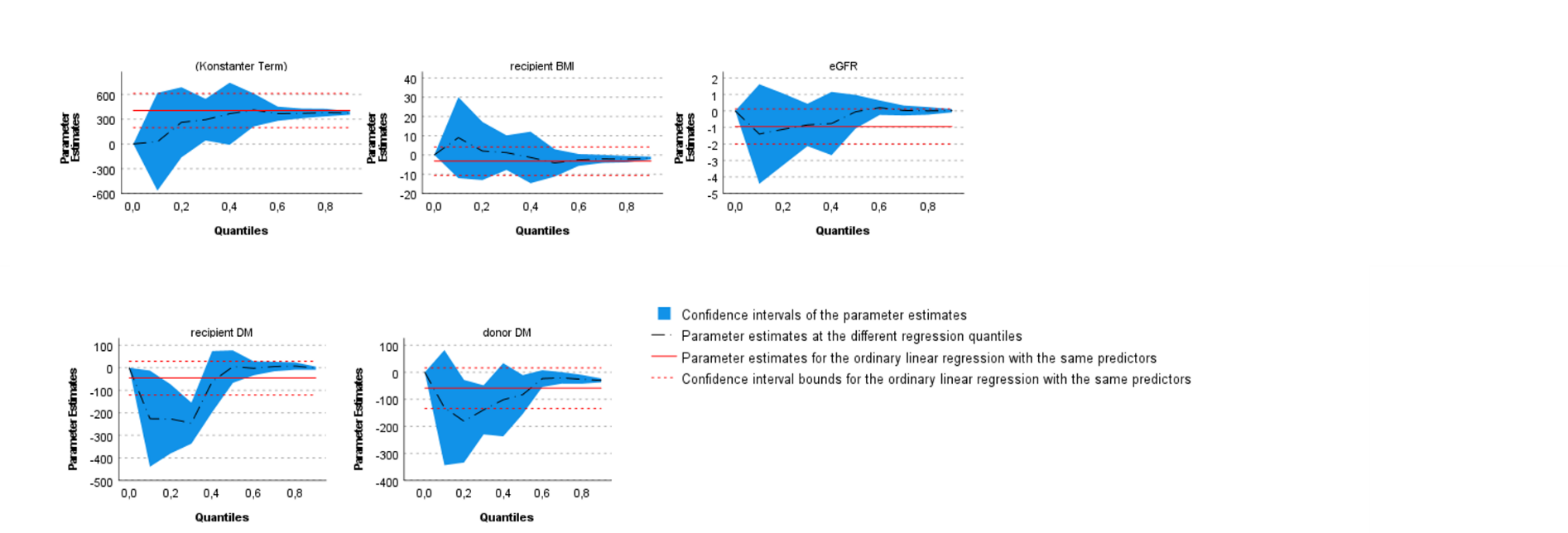


**Legend:** The figure shows the influence of preoperative variables on DAOH in a multivariable quantile regression model. Y-axis shows DAOH estimates while X-axis shows different quantiles. The black line represents parameter estimates at different regression quantiles. The Confidence intervals of quantile regression are presented in blue. Red lines represent parameter estimates and confidence interval of an ordinary linear regression with same variables. Amongst these parameters, donor diabetes mellitus (DM) and recipient diabetes mellitus showed significant association with DAOH in the lower quantiles (10th and 20th percentile). However, model performance was poor with a maximum pseudo-R² of 0.21 in the 20th percentile.

**Figure S4: Influence of additional variables on days out of hospital**


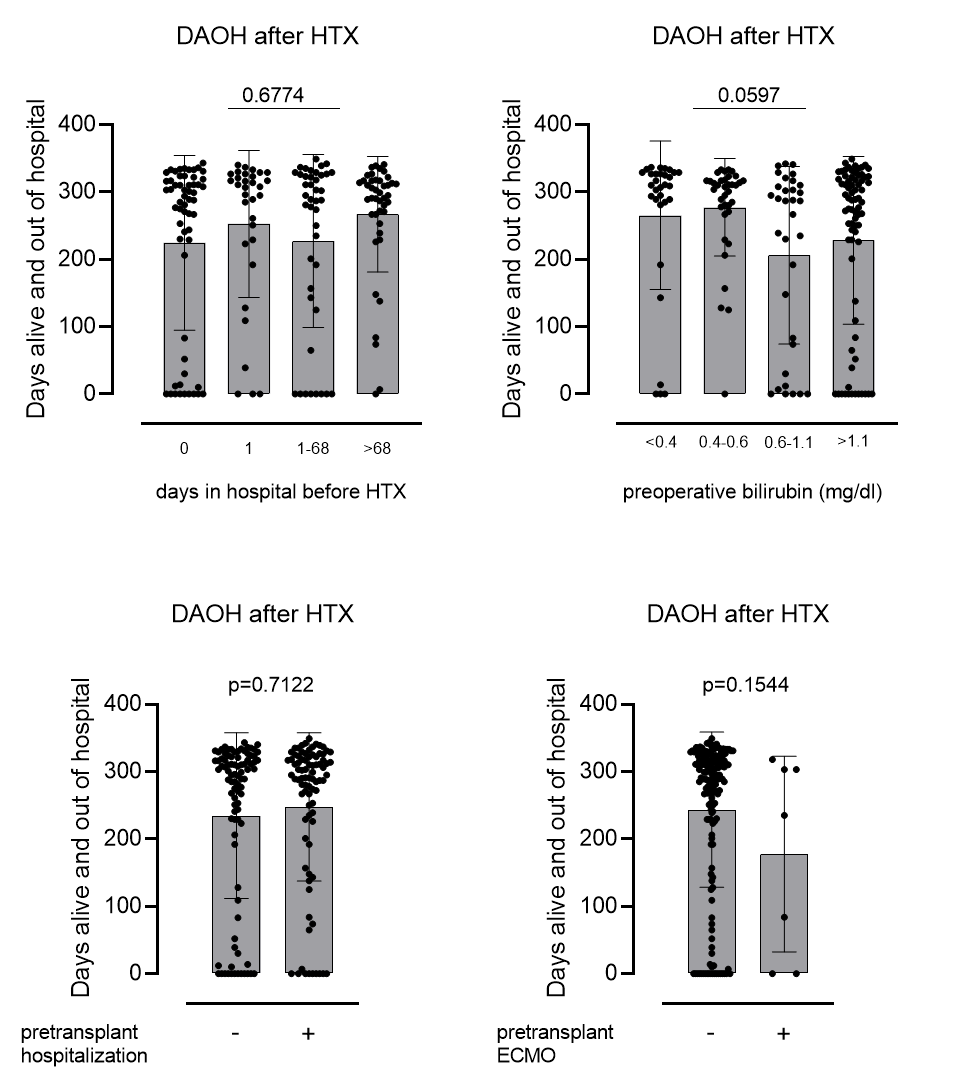


**Legend:** The figure shows univariate analysis of four additional variables and their association with days alive and out of hospital.
